# An Optical Fiber Chemical Sensor for the Detection of Copper(II) in Drinking Water †

**DOI:** 10.3390/s19235246

**Published:** 2019-11-28

**Authors:** Maria Pesavento, Antonella Profumo, Daniele Merli, Lucia Cucca, Luigi Zeni, Nunzio Cennamo

**Affiliations:** 1 Department of Chemistry, University of Pavia, via Taramelli 12, 27100 Pavia, Italy; maria.pesavento@unipv.it (M.P.); antonella.profumo@unipv.it (A.P.); daniele.merli@unipv.it (D.M.); lucia.cucca@unipv.it (L.C.); 2 Department of Engineering, University of Campania Luigi Vanvitelli, Via Roma 29, 81031 Aversa, Italy; luigi.zeni@unicampania.it

**Keywords:** Optical chemical sensors, surface plasmon resonance, human health, copper(II), drinking water

## Abstract

Highly sensitive plasmonic optical fiber platforms combined with receptors have been recently used to obtain selective sensors. A low-cost configuration can be obtained exploiting a D-shaped plastic optical fiber covered with a multilayer sensing surface. The multilayer consists of a gold film, functionalized with a specific receptor, where the surface plasmon resonance (SPR) occurs. The signal is produced by the refractive index variation occurring as a consequence of the receptor-to analyte binding. In this work, a selective sensor for copper(II) detection in drinking water, exploiting a self-assembled monolayer (SAM) of d,l-penicillamine as the sensing layer, has been developed and tested. Different concentrations of copper(II) in NaCl 0.1 M solutions at different pH values and in a real matrix (drinking water) have been considered. The results show that the sensor is able to sense copper(II) at concentrations ranging from 4 × 10^−6^ M to 2 × 10^−4^ M. The use of this optical chemical sensor is a very attractive perspective for fast, in situ and low-cost detection of Cu(II) in drinking water for human health concerns. Furthermore, the possibility of remote control is feasible as well, because optical fibers are employed.

## 1. Introduction

Copper(II) is an essential element for human health since it is a cofactor of many enzymes involved in chemical redox reactions, with antioxidant activity. Thus, for example the USA and Canada established a recommended dietary allowance (RDA) for adults of 900 μg/day. On the other hand, it can be of concern for human health when present at high concentration, as it is also able to induce oxidative stress by different mechanisms [1]. Actually, it appears to be involved in neurological disorders of high social concern as Parkinson and Alzheimer disease [2,3]. In the environment, copper(II) is mainly present in soil components, as clays, minerals and organic solids (for example humic substances), due to the low solubility of many of its compounds and their easy adsorption. For the above reasons copper(II) is usually present at low concentration in natural waters, but it can be at relatively high concentrations in drinking water at the tap. In fact, copper(II) concentration in drinking water may increase during distribution, especially in waters with an acid pH or with a high carbonate content at alkaline pH (US EPA, 1995), due to the corrosion of copper plumbing. This seems to be the major source of copper in drinking water (US EPA,1991; Health Canada, 1992; IPCS, 1998; US NRC, 2000) even if copper is widely diffused in the environment, having a large number of everyday life applications. 

Many investigations from Europe, Canada and USA indicate that copper levels in drinking water can range from 0.005 to >30 mg/L. At a concentration greater than 6 mg/L, it can have some detrimental effects on health causing nausea, vomiting, abdominal pain and cramps, headache, dizziness, weakness, and diarrhea [4]. From experiments on human volunteers, it has been found that some symptoms appeared for copper(II) concentration in drinking water as low as 2 mg/L [4]. A guideline value for the maximum concentration in drinking water has been established at 2 mg/L level.

Notice that most people consume less than recommended amounts of copper in their diet, however symptoms of copper deficiency are relatively rare [4]. For this reason, in recent years more concern has been raised about copper toxicity than copper deficiency.

The most important analytical methods for the detection of copper in water are atomic absorption spectrometry (AAS) with flame detection, graphite furnace atomic absorption spectroscopy, inductively coupled plasma atomic emission spectroscopy, inductively coupled plasma mass spectrometry (ICP-MS) and stabilized temperature platform graphite furnace atomic absorption (ISO, 1986, 1996; ASTM, 1992, 1994; US EPA, 1994). The ICP-MS technique has the lowest detection limit (0.02 μg/L), while detection limits for the other techniques range from 0.7 to 3 μg/L or even 20 μg/L. The US EPA (1991) has established 50 μg/L as the practical quantification limit for analytical methods for copper determination in drinking water. 

Sensing methods for detection and analysis of copper(II) in drinking waters are of great help for a better quality control directly at the tap. Actually, a large number of sensing devices have been proposed not only for the determination of copper(II) content, but also for that of other potentially toxic metal ions. Different transduction methods have been considered, in particular, electrochemical sensing has been widely investigated, often taking advantage of novel nanostructured materials and of the recently introduced miniaturized screen-printed cells, as reported in several reviews [5,6,7]. Optical transduction for heavy metal ions detection has been proposed too, including several surface plasmon resonance (SPR) or localized SPR (LSPR) based sensing devices, as it has been recently reviewed [8,9,10]. SPR is an efficient marker-free method for the detection and investigation of the interaction between an immobilized receptor and a substrate, being highly sensitive, selective, fast, and cost effective. It has been widely proposed for detection of different substances, including various metal ions [11,12,13], for a couple of decades. The detection relies on the variation of the refractive index of the receptor layer in tight contact with the metal surface, at which the SPR is excited when the metal ion combines with the receptor.

In a large number of previous papers, the SPR sensing of heavy metal ions relied on the Kretschmann configuration, which is not suitable for out-of-the-lab determinations. A few papers have been recently presented, dealing with sensors for heavy metal ions based on SPR or LSPR transduction in which the plasmonic phenomenon was detected by optical fibers (OF) using a spectral interrogation, i.e., based on the resonance wavelength variation [9,14,15]. The sensing layer was often a thin film, a few nm thick, of a composite material which usually presented a certain selectivity towards selected metal ions. 

We have recently developed a very convenient experimental setup for SPR sensing of metal ions, based on a multimode plastic optical fiber (POF) [15]. The receptor was a specific complexing agent of Fe(III), deferoxamine, which was deposited as a self-assembled monolayer on the gold thin layer at which the SPR takes place. The sensing platform was built up directly on the POF, where half of the cladding was eliminated by mechanical polishing, obtaining a typical D-shaped profile [16]. The sensing multilayer was deposited on the D-shaped area of the POF. The use of OFs for plasmon excitation is favorable because of the low-cost and low dimension apparatus making it possible a very convenient out-of-the-lab application [17,18,19].

The aim of the present work was the development of an SPR sensor for copper(II) detection in drinking water, based on the D-shaped POF previously proposed for Fe(III) detection (SPR-POF), but exploiting d,l-penicillamine as the receptor. This ligand was considered since it is a strong complexing agent for copper(II), widely used, for example, for therapeutic aims, as in Wilson’s disease [20]. Moreover, it can be easily linked as a self-assembled monolayer to a gold surface taking advantage of the –SH group present in d,l-penicillamine molecule, as demonstrated in the case of a previously proposed electrochemical sensor [21]. Some preliminary results [22] were very promising, so that a systematic investigation on the possibility of applying the proposed SPR sensor in OF to the determination of Cu(II) in real drinking water samples was investigated in the present research.

To date, the proposed chemical optical fiber sensor system has only been used in a laboratory scenario, but it can be directly connected to an online platform, allowing it to store, analyze, and display the sensor’s data, thereby realizing a “smart” low-cost POF Cu(II) sensor. 

## 2. Materials and Methods

All the reagents were purchased from Sigma–Aldrich (Milan, Italy) and used as received, included d,l-Penicillamine. Copper(II) diluted solutions were daily prepared in ultrapure grade water (Milli-Q, Millipore) from a standard 1000 mg/L atomic adsorption Cu(II) solution. 

Refractive index (*n*) of the liquids was checked by an Abbe refractometer (model RMI by Exacta Optech, Germany).

### 2.1. Preparation of the SPR Platform on POF 

**Figure 1:**
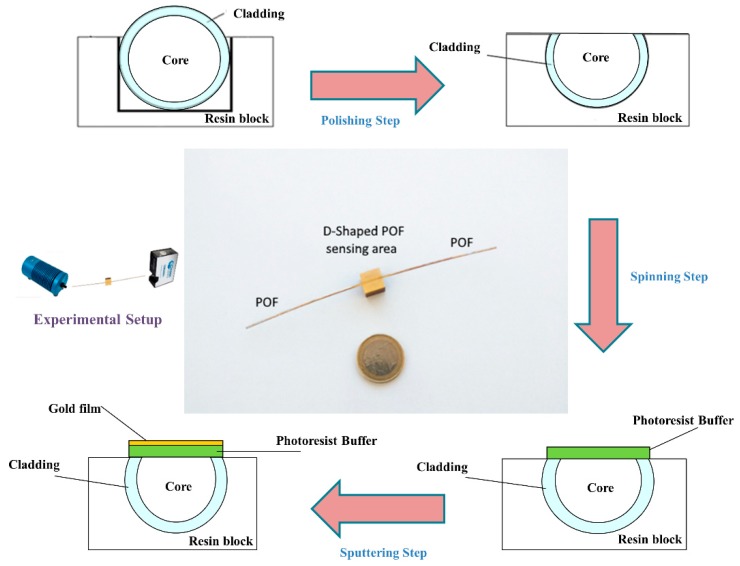
Surface plasmon resonance (SPR) sensor platform in plastic optical fiber (POF) (SPR-POF) and an outline of the three production steps.

A polymer optical fiber with a core of poly-methylmethacrylate (PMMA) of 980 µm and a cladding of fluorinated polymer of 10 µm (1 mm of total diameter) has been modified to realize the optical sensor platform. It can be obtained in three steps (see outline in Figure 1), as reported in more detail in [16] and briefly described below.

First, a D-shaped region was produced on the POF by removing the cladding and part of the core, along half circumference, by a very simple polishing process based on two kinds of polishing papers [16]. The D-shaped sensing region has a length of 10 mm and a width of about 850 µm.

Second, a Microposit S1813 photoresist was spun (6000 rpm for 60 s) on the exposed POF core with the use of a spin coater machine to obtain a layer between the POF core and the metal. This layer has a refractive index higher than that of the POF core and can considerably improve the performance. The spin coating technique allowed the deposition of a uniform photoresist layer about 1.5 µm thick [16]. 

Finally, a thin gold film was sputtered by a Bal-Tec SCD 500 machine. The sputtering process was repeated three times by applying a current of 60 mA, at 0.05 mbar, for 35 s to obtain a 60 nm thick layer (20 nm of gold for each step). The ligand for copper(II) was immobilized as a monolayer over gold according to the procedure described in the following paragraph. Figure 1 shows a picture of this SPR sensor in POFs, together with an outline of the production process steps. 

### 2.2. Functionalization of the Gold Surface

The gold surface was modified by depositing a self-assembled monolayer of d,l-penicillamine on the gold surface, according to a protocol previously described for a voltammetric sensor [21]. The platform was rapidly flushed with ethanol and dipped in 5 mM ethanolic solution (20% ethanol) of d,l-penicillamine for 12 h. It was then rinsed with ethanol and water before use. Since a monolayer of d,l-penicillamine is formed by the described procedure [21], it is approximately a few nanometers thick. 

SPR phenomenon was used to monitor the functionalization process, by comparing the normalized spectra, as described in the following. 

### 2.3. Experimental Set Up

The experimental apparatus consists of the Halogen lamp HL-2000-LL (manufactured by Ocean Optics, Dunedin, FL, USA) used as white light source, the POF sensor platform (reported in Figure 1), and the spectrometer (FLAME-S-VIS-NIR-ES, manufactured by Ocean Optics, Dunedin, FL, USA) [15,16]. Figure 1 shows a scheme of the experimental setup, too.

The white light source presents an emission range from 360 nm to 1700 nm, whereas the spectrometer has a detection range from 350 nm to 1023 nm. The transmission spectra, along with data values, were displayed on-line on the computer screen and saved by Spectra Suite software (Ocean Optics, Dunedin, FL, USA).

### 2.4. Binding Measurements

All the experiments were performed using the same procedure. About 50 µl of the solution, with copper(II) at different concentrations (both in standard solution or in real matrix), were dropped over the sensing region of the POF and incubated at room temperature for ten minutes. At the end of this incubation time, the spectrum was registered and saved. The plasmonic transmission spectra were obtained by the Matlab software (MathWorks, Natick, MA, USA) using, as reference for normalization, the spectrum acquired with air as surrounding medium over the gold surface derivatized with d,l-penicillamine [15,16]. The Hill fittings of the experimental data were obtained by OriginPro software (Origin Lab. Corp., Northampton, MA, USA).

The binding between the chemical receptor, immobilized on gold surface on the POF platform, and the analyte (Cu(II)) was evaluated for concentration ranging from 0.0 to 1.5 10^−4^ M.

### 2.5. Binding Evaluation and Standardization

The data (resonance wavelength variation with respect to the resonance wavelength of the blank solution Δλ) vs. copper(II) concentration were fitted by the Hill equation [23], using the software OriginPro:

(1) $$Y=Y_{max}\frac{c}{K+c}$$

where *Y* is the concentration of the analyte in the layer near the transductor (in mmol/g), *Y*_max_ is the same value at saturation (mmol/g), *c* is the concentration of the analyte in the solution phase (M), and *K* is a constant value (M). It has been assumed that the third parameter of the Hill equation in OriginPro, *n*, is equal to 1. 

Considering the Langmuir isotherm as the model for the adsorption on the ligand monolayer, the following relationship holds:

(2) $$Y=Y_{max}\frac{K_{aff}c}{1+K_{aff}c}$$

where *K*_aff_ is the reciprocal of the parameter *K* in the Hill equation. 

When only the signal (Δλ) directly proportional to Y, is measured, the Langmuir isotherm is as follows:

(3) $$\Delta \lambda_{c\;}=\;\lambda_{c\;}-\;\lambda_{0\;}=\;\Delta \lambda_{max\;}\cdot \frac{c}{K+\;c}$$

where Δλ_max_ is the value of the maximum resonance wavelength variation at increasing concentration of Cu(II), i.e., the value at saturation. The symbol λ_c_ indicates the resonance wavelength at *c* concentration. The resonance at 0 M concentration (blank) is indicated as λ_0_, whereas *K* is the Hill’s parameter, corresponding to the reciprocal of K_aff_ in the Langmuir model. Relationship (3) is used as the standardization curve.

## 3. Results and Discussion

### 3.1. Functionalization of the Gold Surface of the POF Sensor

**Figure 2:**
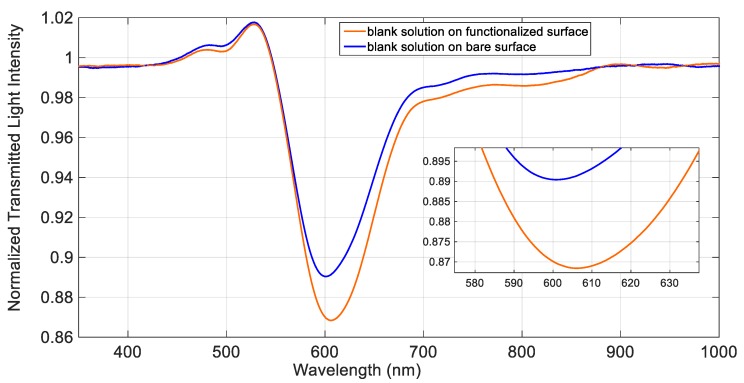
SPR spectra obtained before (blue line) and after (red line) the functionalization process, in aqueous NaCl 0.1 M.

Figure 2 shows the SPR spectra obtained in NaCl 0.1 M water solution (blank solution, which has a refractive index value very similar to that of pure water), before and after the deposition of the d,l-penicillamine monolayer. As it is evident from Figure 2, in presence of the same bulk solution, the resonance wavelength is shifted to higher values after the functionalization process, confirming that the receptor was successfully immobilized on the gold surface. Indeed, when molecules are immobilized on the gold surface, even as a thin layer as a self-assembled monolayer (SAM), an increase of the refractive index occurs, determining a shift of the resonance wavelength to higher values. A typical shift (Δλ), associated to a self-assembled monolayer on an SPR-POF platform of this kind, ranges from 5 nm to 20 nm, as previously found, for example, in the case of an aptasensor [24]. In the present case, a shift of about 6 nm has been obtained after the deposition of the d,l-penicillamine monolayer on gold (see Figure 2). A small enlargement of the peak width was observed in the derivatized platform with respect to the bare one. 

### 3.2. Cu(II) Detection in Solutions at Different Acidities

**Figure 3:**
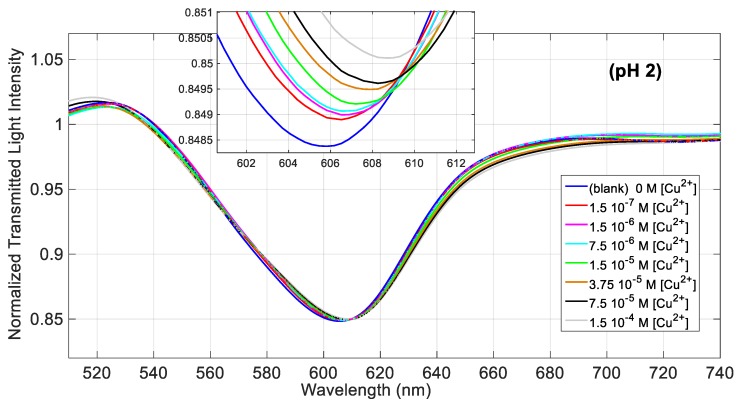
SPR spectra obtained by the SPR-POF sensor at different concentrations of Cu(II) in solution 0.1 M NaCl at pH = 2, over the concentration range from 0 M to 1.5 × 10^−4^ M.

The binding of copper(II) to the SPR sensor has been investigated in 0.1 M NaCl solutions at different pHs, which, for example, include the acidity of some common beverages. Figure 3 shows the transmission spectra of the SPR-POF sensor, normalized to the reference spectrum (the spectrum acquired in air), obtained at increasing concentrations of Cu(II) in NaCl 0.1 M solution at pH = 2. By increasing the concentration of the analyte, the resonance wavelength is shifted to higher values, indicating that the metal ion binds to d,l-penicillamine linked to the gold surface even at this very low acidity, causing an increase of the refractive index of the receptor layer. The shape of the spectrum is similar to that obtained in the preliminary investigation [22] while the resonance wavelength (606 nm) is slightly higher than 604 nm [22], obtained however with a different sensor and at a different pH.

**Figure 4:**
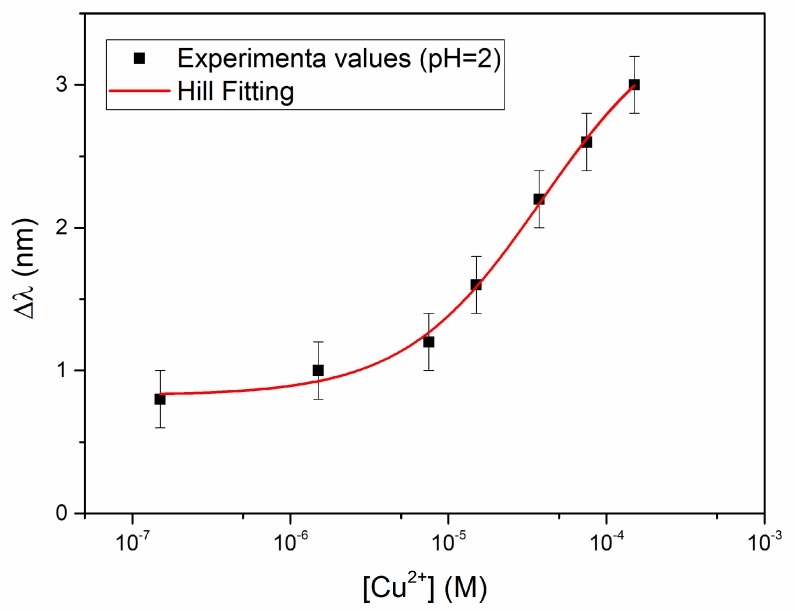
Plasmon resonance wavelength shift (Δλ) vs. concentration of Cu(II) in buffer solution at pH = 2, in semi-log scale, with error bars and Hill fitting to the experimental data.

Figure 4 reports the resonance wavelength variation with respect to the blank, Δλ (nm), as a function of the analyte concentration, in a semi-log scale, together with the error bars and the Hill fitting according to Equation (3).

In Figure 4 each experimental point (black square) is the average of three subsequent measurements and the error bars represent the max measured standard deviation (0.2 nm). The relationship between the signal, i.e., the variation of the resonance wavelength Δλ, and the concentration of Cu(II) is hyperbolic, so that the Hill equation has been assumed for fitting, as obtained by OriginPro Software (OriginPro8.5, Origin Lab. Corp. Northampton, MA, USA), assuming for convenience that *n* = 1. The parameters evaluated by the Hill equation are reported in Table 1.

The reduced chi-square value indicates that fitting is not completely satisfactory, since probably the error variance is underestimated by the model, nevertheless the equation can be considered as adequate for analytical application, considering the R-square value. Thus, the obtained parameters can be used for the evaluation of the unknown concentration from the experimentally found Δλ, according to Equation (3). The lower detection limit (LOD), calculated as the ratio of three times the standard deviation of the blank (Δλ_0_) to the sensitivity at low concentration (Δλ_max_/*K*) is 8.8 × 10^−6^ M. Both the sensitivity at low concentration and the LOD, at the conditions here considered, i.e., NaCl 0.1 M at pH = 2, are reported at the end (in Table 5) for the sake of comparison with the other results, together with the affinity constant (1/K = Kaff), which is relevant to characterize the adsorption of copper(II) on the receptor layer. At the end (in Table 5), they are compared to the same parameters obtained at pH near neutrality and in the natural water sample. 

The Δλ_max_ value, here obtained (3.56 nm), is lower than that obtained in the preliminary investigation [22], which was about 6 nm. This is ascribable mainly to the fact that a different photoresist was here used, as the buffer layer, to build the sensor. It has been shown [25] that the S1813 photoresist here used produces an optical sensitivity about one half than that of the photoresist used in the preliminary investigation [22], at the considered refractive index/resonance wavelength.

**Figure 5:**
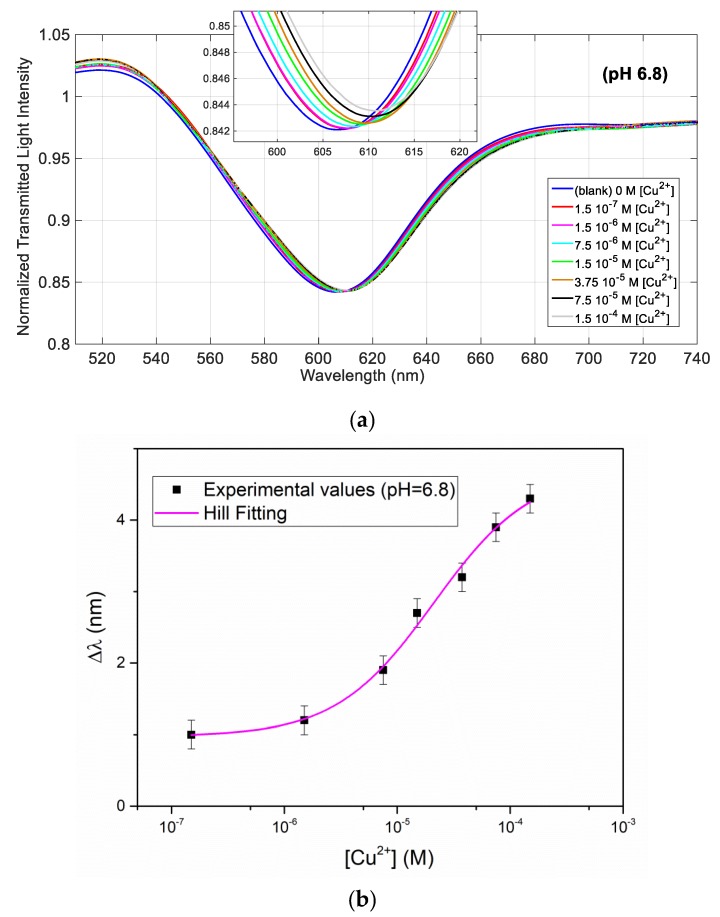
(**a**) SPR spectra obtained by SPR-POF sensor at different concentrations of Cu(II) in NaCl 0.1 M solution at pH = 6.8, in the range from 0 M to 1.5 × 10^−4^ M. (**b**) Resonance wavelength shift (Δλ) vs. concentration of Cu(II) in semi-log scale, with error bars and Hill fitting.

In order to investigate the effect of the solution pH on the binding parameters, the same experiment has been performed at pH = 6.8. For better comparison, we have used the same concentrations of Cu(II), reported in Figure 3 and Figure 4 for a solution at pH = 2. Figure 5a shows the normalized transmission spectra obtained at different Cu(II) concentrations in a solution at pH = 6.8, whereas Figure 5b shows the resonance wavelength variation as a function of the Cu(II) concentration, in a semi-log scale, with the error bars and the Hill fitting. 

The Hill fitting parameters (evaluated considering *n* = 1), are reported in Table 2, whereas the affinity constant (*K*_aff_), the sensitivity at low concentration, and LOD of Cu(II) detection are reported at the end (in Table 5). 

The fitting by the Hill equation is acceptable at this pH too. The parameters are similar at the two considered acidities, however *K*_aff_ is about twice higher at pH = 6.8 than at acidic pH, with a slightly lower LOD. Actually, the receptor ligand d,l-penicillamine contains an aminoacidic moiety, thus it is expected that the acidity may have an influence. Protons can be considered as competitors of the copper(II) ions for the binding to the ligand, so influencing the (apparent) affinity constant. Since the protonation constants of d,l-penicillamine are log*K*_1_ = 10.65, log*K*_2_ = 7.97 and log*K*_3_ = 1.94 [26] it is expected that, at the considered acidities, the protonation degree is different. From the protonation constants, it can be evaluated that at pH = 6.8 d,l-penicillamine in solution is in the biprotonated form at 93.6%, probably as a neutral zwitterion, while at 53% at pH around 2. The situation could be different when d,l-penicillamine is linked to the solid phase, since the thiolic group is near to the gold surface, and is probably not easily accessible to the protonation. In any case, the experimentally found difference of *K*_aff_ for copper(II), at the two considered acidities, is low, corresponding to the prediction made on the basis of the protonation constants of d,l-penicillamine in solution phase. This is confirmed by the fact that the resonance wavelength is very near at the two considered acidities, indicating that the refractive index of the receptor monolayer is similar.

### 3.3. Cu(II) Detection in Natural Water (Real Matrix)

As an example of a real matrix, a natural water sample has been considered, the chemical composition (main components) of which is reported in Table 3. The natural water sample considered was a hard water containing a high concentration of electrolytes. 

The concentration of Cu(II) originally present was found to be lower than 0.05 mg/L, so that it can be considered as zero. This corresponds to normal levels of copper(II) in natural waters. The sample was measured without any previous treatment, as for example pH or ionic strength adjustment, only known concentrations of copper(II) were added.

**Figure 6:**
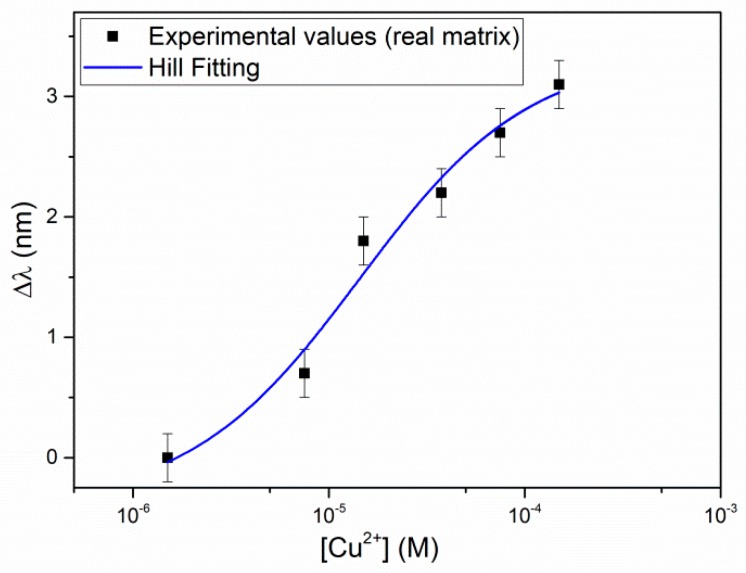
Resonance wavelength shift (Δλ) versus concentration of Cu(II) in a natural water at high salinity, in semi-log scale, with error bars and Hill fitting of data obtained by the SPR-POF sensor.

Figure 6 shows the resonance wavelength variation, Δλ (nm), as a function of the Cu(II) concentration, in a semi-log scale, with the error bars and the Hill fitting. The Hill fitting parameters are listed in Table 4, and the affinity constant (*K*_aff_), the sensitivity at low concentration and the LOD of Cu(II), with the Hill equation approximated with *n* = 1, are reported in Table 5.

The results in natural water are similar to those obtained in NaCl 0.1 M. Despite of the presence of high concentrations of possible interfering ions, as for example, calcium, or of substances able to complex copper(II), as sulphate and/or humic substances, *K*_aff_ is even somewhat higher in the natural water sample than in the buffer and correspondingly, the LOD is somewhat lower. In this case, the detection limit is 0.2 mg/L, which is high for analysis in natural water (see US EPA (1991)) but is suitable for drinking water control, since a maximum limit concentration of 2 mg/L has been recommended for copper(II) in this kind of water.

These results indicate that the selectivity of the proposed sensor in water is very good, considering that the natural sample examined has a high mineral content, for example a calcium concentration as high as 2 × 10^−2^ M (see Table 3). This confirms some previously reported preliminary results, showing that the resonance wavelength of the SPR-POF sensor with d,l-penicillamine SAM is not affected by low concentrations of metal ions as Ca(II) or Fe(III) [22].

The parameters characterizing the adsorption on the d,l-penicillamine SAM and the sensor performances, are compared in Table 5. The sensitivity at low concentration is the slope of the straight line obtained from Equation (3), when *c* is much lower than *K* (or 1/*K*_aff_). LOD has been evaluated as three times the standard deviation of Δλ_0_, i.e., the standard deviation of the solution with *c* = 0 M, as obtained from the Hill fitting (second column in Table 1, Table 2 and Table 4).

### 3.4. Discussion

In literature, several Cu(II) sensors have been proposed based on different receptor layers, some examples of which are reported in Table 6, with the detection range and the receptor. It must be noticed that all the reported samples are SPR sensors based on the Kretschmann configuration. Some SPR sensors, based on OFs, have been reported too in the previous literature, but not for copper(II) determination. 

As seen by comparison with the results in Table 6, the performances obtained in this work, exploiting a very simple and low-cost sensor based on POF, are very similar to those obtained by more expensive sensor configurations. In some cases, the LOD is lower than that of the sensor proposed in the present work, but this is not strictly required for the detection in drinking water. In this particular application field, a very low detection limit is not necessary. However, a reduced LOD could be obtained, if required, by exploiting the sensing approach based on POF here proposed but with some simple modifications of the experimental configuration. For example, in some previously published papers, we demonstrated that the LOD could be reduced by implementing tapered POFs, as for l-nicotine detection [27], or tapered POFs combined with a sensing layer obtained by gold nano-stars suspended in MIPs, as tested for trinitrotoluene detection [28]. 

Replacing a typical Kretschmann configuration with plastic optical fibers makes it possible to reduce the sensor cost and dimensions, with the possibility to integrate the SPR sensing platform in the telecommunication systems for remote and on-site measurements.

## 4. Conclusions

In this investigation, the response of an SPR chemical sensor, based on POFs, to copper(II) in synthetic solution of different acidity and in a natural water of high mineral content, has been investigated. We demonstrated that the low-cost, portable, and small size sensor here proposed is sufficiently sensitive and selective to be used for remote and on-site determination of Cu(II) content in drinking water, at concentration levels of interest for human consumption in view of the health concern about possible toxic metal ions intake. 

The future development of this chemical POF sensor system will require significant advances towards the miniaturization and full integration of the POF platform with optoelectronics devices, to obtain an inexpensive “smart” sensor system, with a small size, and the possibility of internet connection (exploiting the capability of the so-called “Internet of Things”).

## Figures and Tables

**Table 1:** Hill parameters of Cu(II) detection in NaCl 0.1 M solution at pH = 2, with *n* = 1. SE is the standard error of the value.

Δλ_0_ (nm)		Δλ_max_ (nm)		*K* (M)		*n*		Statistics	
Value	SE	Value	SE	Value	SE	Value	SE	Reduced chi-Square	R-Square
0.82	0.27	3.56	0.78	3.89 × 10^−5^	3.43 × 10^−5^	1	0	3.46	0.87

**Table 2:** Hill parameters of Cu(II) detection in in NaCl 0.1 M solution at pH = 6.8, with *n* = 1. SE is the standard error of the value.

Δλ_0_ (nm)		Δλ_max_ (nm)		K (M)		*n*		Statistics	
Value	SE	Value	SE	Value	SE	Value	SE	Reduced Chi-Square	Adj. R-Square
0.96	0.35	4.71	0.61	2.10 × 10^−5^	1.33 × 10^−5^	1	0	5.00	0.91

**Table 3:** Composition of the natural water (real matrix) considered for sensing Cu(II).

Parameter			Value
pH			7.1
Specific electrical conductivity at 20 °C		µS/cm	2300
fixed residue at 180 °C		mg/L	2493
bicarbonate	HCO_3_^−^	mg/L	288
chloride	Cl^−^	mg/L	8.5
sulphate	SO_4_^2-^	mg/L	1604
sodium	Na^+^	mg/L	9.0
potassium	K^+^	mg/L	2.6
calcium	Ca^2+^	mg/L	602
magnesium	Mg^2+^	mg/L	92.0
strontium	Sr^2+^	mg/L	11.8
dissolved iron	Fe	mg/L	2.1
Boron	B	mg/L	0.03
lithium	Li^+^	mg/L	0.01
nickel	Ni	mg/L	0.005
copper(II)	Cu^2+^	mg/L	<0.05

**Table 4:** Hill parameters of Cu(II) detection in natural water (real matrix). SE is the standard error of the value.

Δλ_0_ (nm)		Δλ_max_ (nm)		K (M)		*n*		Statistics	
Value	SE	Value	SE	Value	SE	Value	SE	Reduced Chi-Square	Adj. R-Square
–0.39	0.30	3.35	0.25	1.43 × 10^−5^	5.05 × 10^−6^	1	0	1.14	0.97

**Table 5:** Comparison of the parameters for Cu(II) detection in NaCl 0.1 M solutions at different pH and in natural water at pH = 7.1.

Parameters	Value (pH = 2)	Value (pH = 6.8)	Value (Natural Water)
*K_aff_* (M^−1^) (*K_aff_* = 1/*K*)	2.57 × 10^4^	4.74 × 10^4^	6.99 × 10^4^
Sensitivity at low *c* (nm/M) (Sensitivity at low *c* = *Δλ*_max_/*K*)	9.15 × 10^4^	2.24 × 10^5^	2.35 × 10^5^
LOD (M) (3*standard deviation of △λ_0_/Sensitivity at low *c*)	8.85 × 10^−6^	4.78 × 10^−6^	3.84 × 10^−6^

**Table 6:** SPR sensors for Cu(II) detection.

Detection Range	Receptor	Reference
3.9 µM–0.1 mM	Self-assembled monolayer (SAM) d,l-penicillamine	This work
0.1 μM–1.0 mM	2-aminoethane thiolhydrochloride and 6-aminohexane thiolhydrochloride	[29]
0.15mM–7,9 μM	hexadecyltrimethylammonium bromide/nanocrystalline cellulose/graphene oxide	[13]
7.9 μM–1.6 mM	MMW chitosan (glutaraldehyde-crosslinked)	[30]
1.5 μM–not given	Schiff base derivative	[31]
1.6 nM–1.6 μM	Mercury-free gold electrode (SPR combined with ASV)	[32]
1.0 pM–10 mM	Squarylium dye containing polymeric thin-film	[33]
